# Endomicroscopy of the human cochlea using a micro-optical coherence tomography catheter

**DOI:** 10.1038/s41598-021-95991-8

**Published:** 2021-09-09

**Authors:** Janani S. Iyer, Biwei Yin, Konstantina M. Stankovic, Guillermo J. Tearney

**Affiliations:** 1 Department of Otolaryngology – Head and Neck Surgery, Massachusetts Eye and Ear and Harvard Medical School, 243 Charles St, Boston, MA 02114 USA; 2 Program in Speech and Hearing Bioscience and Technology, Harvard University Graduate School of Arts and Sciences, 1350 Massachusetts Ave, Cambridge, MA 02138 USA; 3 Wellman Center for Photomedicine, Massachusetts General Hospital, Boston, MA 02114 USA; 4 Department of Pathology, Massachusetts General Hospital, Boston, MA 02114 USA; 5 Department of Otolaryngology – Head and Neck Surgery, Stanford University School of Medicine, Stanford, CA 94503 USA; 6 Department of Otolaryngology – Head and Neck Surgery, Massachusetts Eye and Ear and Harvard Medical School, 801 Welch Road, Stanford, CA 94305 USA

**Keywords:** Cochlea, Microscopy

## Abstract

Sensorineural hearing loss (SNHL) is one of the most profound public health concerns of the modern era, affecting 466 million people today, and projected to affect 900 million by the year 2050. Advances in both diagnostics and therapeutics for SNHL have been impeded by the human cochlea’s inaccessibility for in vivo imaging, resulting from its extremely small size, convoluted coiled configuration, fragility, and deep encasement in dense bone. Here, we develop and demonstrate the ability of a sub-millimeter-diameter, flexible endoscopic probe interfaced with a micro-optical coherence tomography (μOCT) imaging system to enable micron-scale imaging of the inner ear’s sensory epithelium in cadaveric human inner ears.

## Introduction

**Figure 1:**
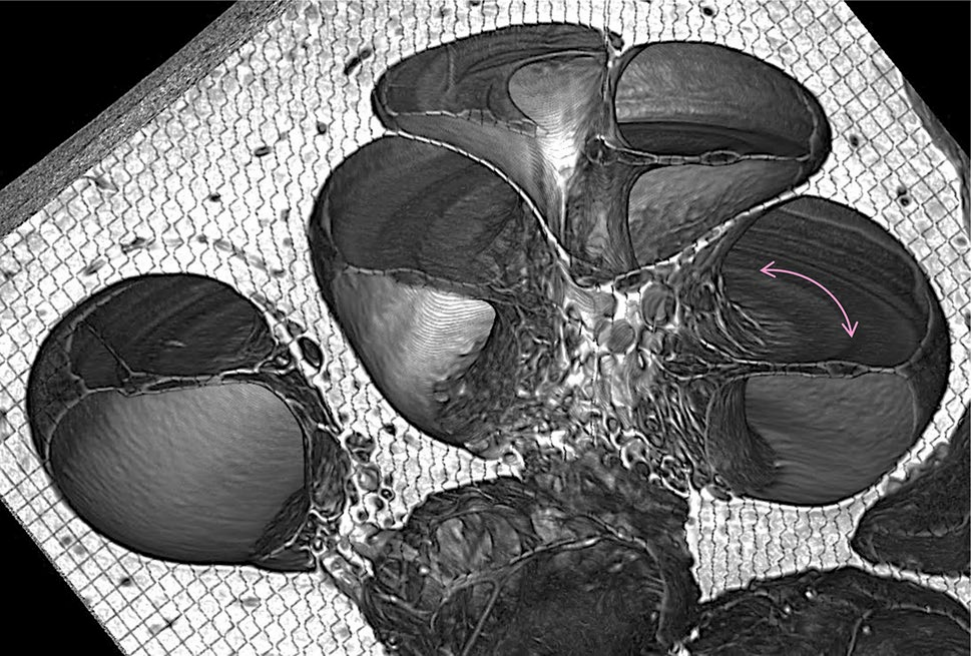
Virtual mid-modiolar cross-section through a human cochlea imaged using synchrotron radiation phase contrast imaging (SR-PCI). The location and orientation of the organ of Corti are indicated with the magenta arrow.

When a sound pressure wave enters the ear canal and interacts with the middle ear’s ossicular chain of bones, it transforms into a complex and highly sophisticated pattern of mechanical vibrations that is ultimately detected, transduced, and delivered to the auditory nerve by the cochlea, the sensory organ that facilitates hearing (Fig. 1). The cochlea’s sensory epithelium, the organ of Corti, comprises a mosaic of micron-sized cells and nerve fibers that selectively respond to different sound frequencies based on their location along the length of the spiraling epithelium. Studies conducted in animal models and histologically-prepared post-mortem human cochlear specimens suggest that age-, drug-, and noise-induced SNHL are caused by damage to the organ of Corti’s composite structures^1,2^; these structures are thus the targets for developing drug and gene therapies for SNHL^1^.

Clinical imaging techniques for human cochlear evaluation have historically failed at resolving the aforementioned cells and nerve fibers that are implicated in SNHL. Specifically, high-resolution computed tomography (CT) and magnetic resonance imaging (MRI) are incapable of providing cellular-level information about the organ of Corti’s integrity in hearing-impaired patients, precluding objective diagnosis of the pathologic causes of hearing loss. In addition, the lack of a clinical intracochlear imaging technique prevents otologists from positioning electrode arrays during cochlear implantation with specificity and consistency; indeed, blind electrode array insertion may in part explain the high variability in hearing outcomes for cochlear implant patients^3,4^.

To address these clinical needs, we developed a sub-millimeter diameter, flexible endomicroscopic probe, interfaced it with a system that performs μOCT imaging with micron-scale resolution, and tested its ability to image the organ of Corti from within the cochlea in intact cadaveric human temporal bone specimens. μOCT is a high-resolution descendant of optical coherence tomography (OCT), a cross-sectional imaging technique that generates two- and three-dimensional images by measuring properties of the back-scattered or back-reflected light from different depths within a biological tissue. OCT has achieved recent attention and popularity for its diverse range of clinical applications^5–7^, and it is particularly well-suited for intracochlear imaging because of its minimally invasive nature, its ability to afford micron-scale resolution, and the ease with which it can be interfaced with endoscopes to access the body’s interior^5^.

## Results

**Figure 2:**
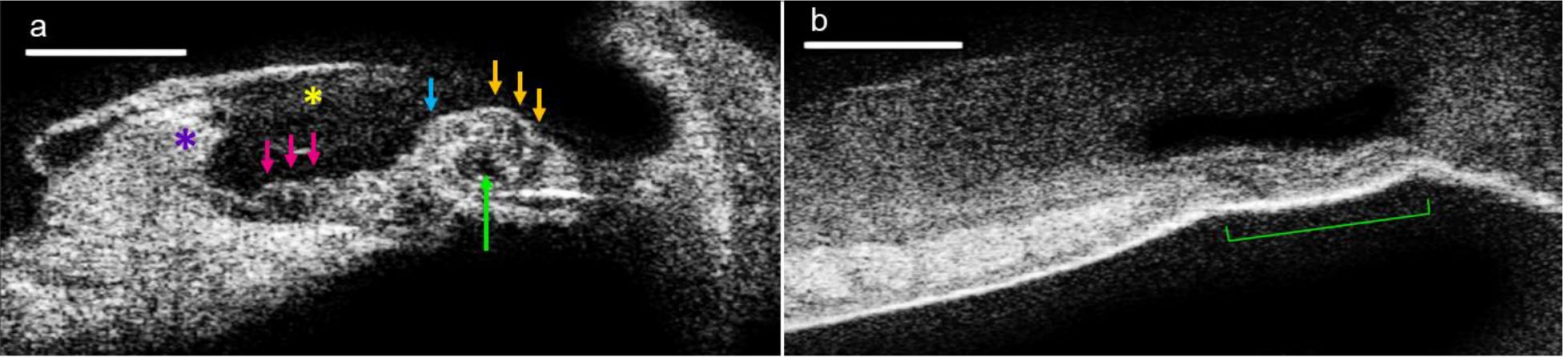
Mouse organs of Corti imaged using benchtop μOCT. (**a**) Organ of Corti dissected from the basal cochlear turn of a healthy mouse. Purple = spiral limbus; yellow = tectorial membrane; Arrows point to structures that are consistent with supporting and/or hair cells as evidenced by visualization of cell membranes. The ability to approximate cell type is given by their anatomical location: Pink, supporting cells; blue, inner hair cell; orange, outer hair cells. The green arrow demarcates the tunnel of Corti. (**b**) Organ of Corti dissected from the basal cochlear turn of a noise- exposed mouse. Flat epithelium morphology is marked with a green bracket. Scales = 100 μm.

The present effort was inspired by our group’s demonstrations of μOCT’s ability to (a) enable visualization of individual cells and nerve fibers in the guinea pig organ of Corti in situ^8^, (b) distinguish between healthy and noise-damaged mouse organ of Corti in situ (Fig. 2), and (c) allow extended depth-of-focus, micron-scale imaging when interfaced with submillimeter-diameter, flexible endoscopes^9^.

**Figure 3:**
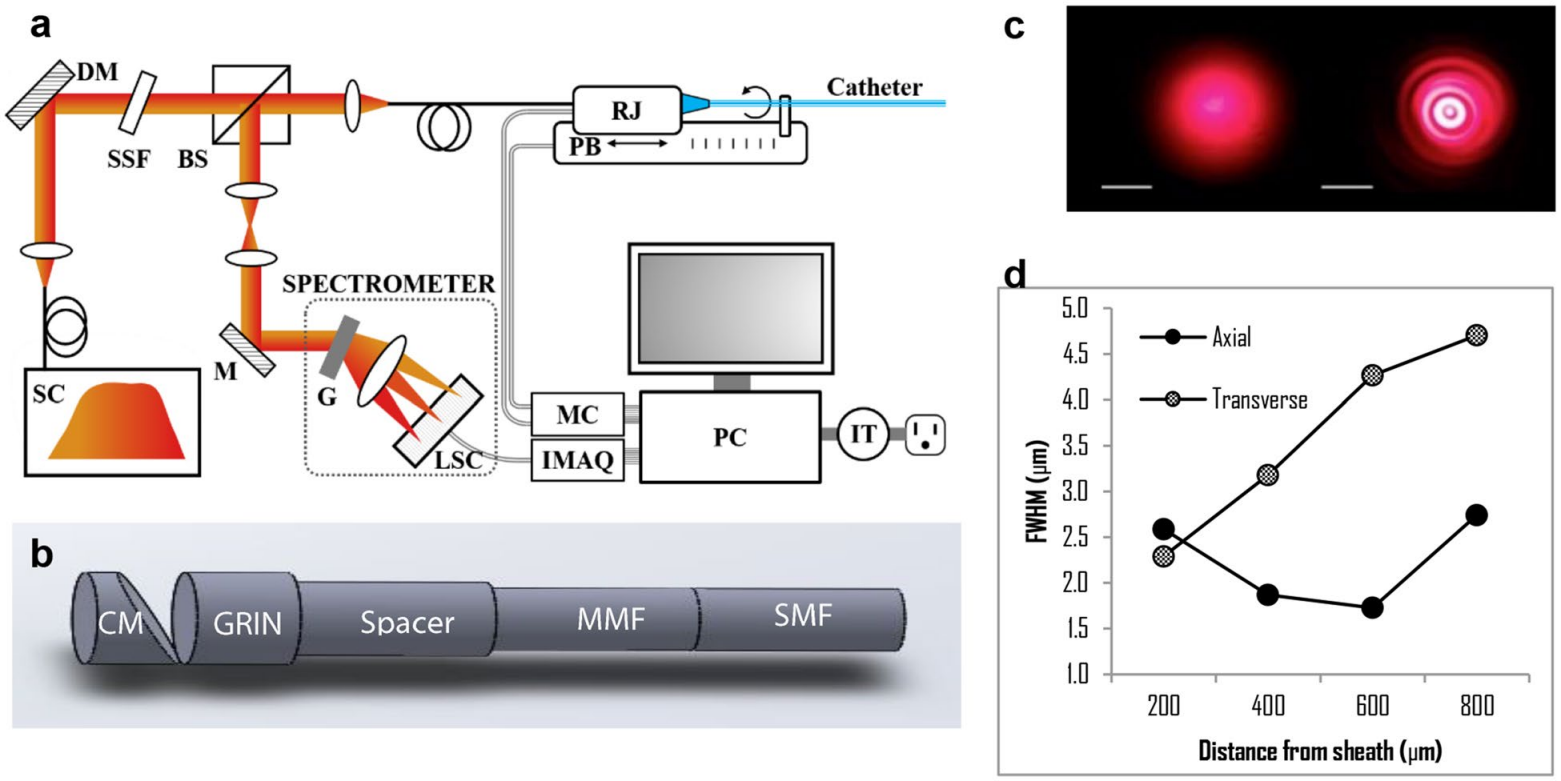
(**a**) Schematic depicting the elements comprising the μOCT system that is interfaced with an endomicroscope for intracochlear imaging. *DM* dichroic mirror, *SSF* spectral shaping filter, *BS* beamsplitter, *PB* pullback stage, *RJ* rotary junction, *SC* supercontinuum laser, *M* mirror, *G* diffraction grating; *LSC* line scan camera, *MC* motor controller, *IMAQ* image acquisition board, *PC* personal computer; *IT* isolation transformer^10^. (**b**) Schematic depiction of the μOCT probe. *CM* cylindrical mirror, *GRIN* graded refractive index lens, *MMF* multimode fiber, *SMF* single mode fiber. (**c**) Plot showing system and probe’s axial and transverse resolution, measured as the full width at half maximum (FWHM) of point spread functions as a function of distance from the probe’s outer sheath. (**d**) Far-field beam output profiles in the transverse plane for Gaussian (left) vs. pseudo-Bessel (right) beams^9^. Scales = 1 cm.

Our spectral domain μOCT system achieves an axial resolution of approximately 1 μm^9,10^. A supercontinuum laser source directs near-infrared light to a beamsplitter, which reflects 10% of the incident light through a common-path fiber optic probe (described below) and ultimately to the tissue; light reflected from the tissue is interfered with reference light to generate an interferogram and is coupled into a custom-made spectrometer (Fig. 3a). A depth-resolved image is reconstructed using standard OCT image reconstruction techniques. This system is interfaced with a self-imaging wavefront division optical probe^10^ that (a) achieves tight, perpendicular focusing via a GRIN lens-mirror complex (Fig. 3b), and (b) extends the imaging depth-of-focus via the addition of a cylindrical waveguide (in this case a multimode fiber element) positioned before the focusing lens (Fig. 3b). The addition of the cylindrical waveguide results in the propagation of multiple co-axially focused pseudo-Bessel fields (Fig. 3c). Light back-reflected from the distal surface of the GRIN lens was leveraged for reference in realizing a common-path interferometry system configuration. Resolution was characterized by scanning the probe over a resolution target at different distances from the probe’s outer sheath. The full width at half maxima (FWHM) of the axial and lateral point spread functions from the resolution target are shown in Fig. 3d.

Circumferential cross-sectional images are acquired via a helical scanning mechanism. The probe is spun inside a transparent sheath such that the light, emitted perpendicular to the probe’s long axis, is directed in a circular pattern. An optical rotary junction couples light from the fixed optical fiber emerging from the μOCT system to the probe’s rotating optical fiber, and the probe’s optical fiber is fixed inside a driveshaft that delivers the torque to the distal end of the probe. The rotary junction was mounted onto a pullback stage that provides axial translation. During experiments, a micromanipulator was used to insert the probe through the cochlea’s round window and into scala tympani, following the insertion procedure that is routine for electrode array insertion during cochlear implantation; real-time imaging was used to locate the basilar membrane and organ of Corti during insertion.

**Figure 4:**
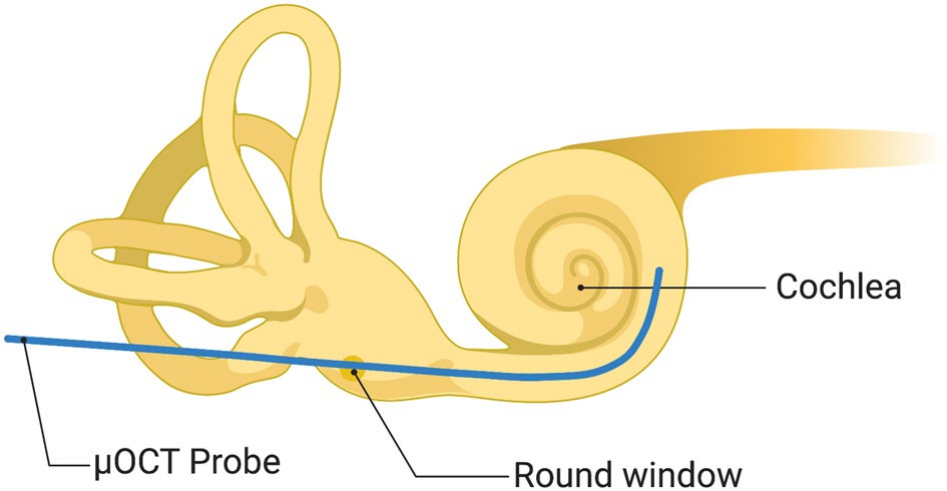
Schematic of the µOCT probe insertion into the cochlea through the round window. Created with www.BioRender.com.

**Figure 5:**
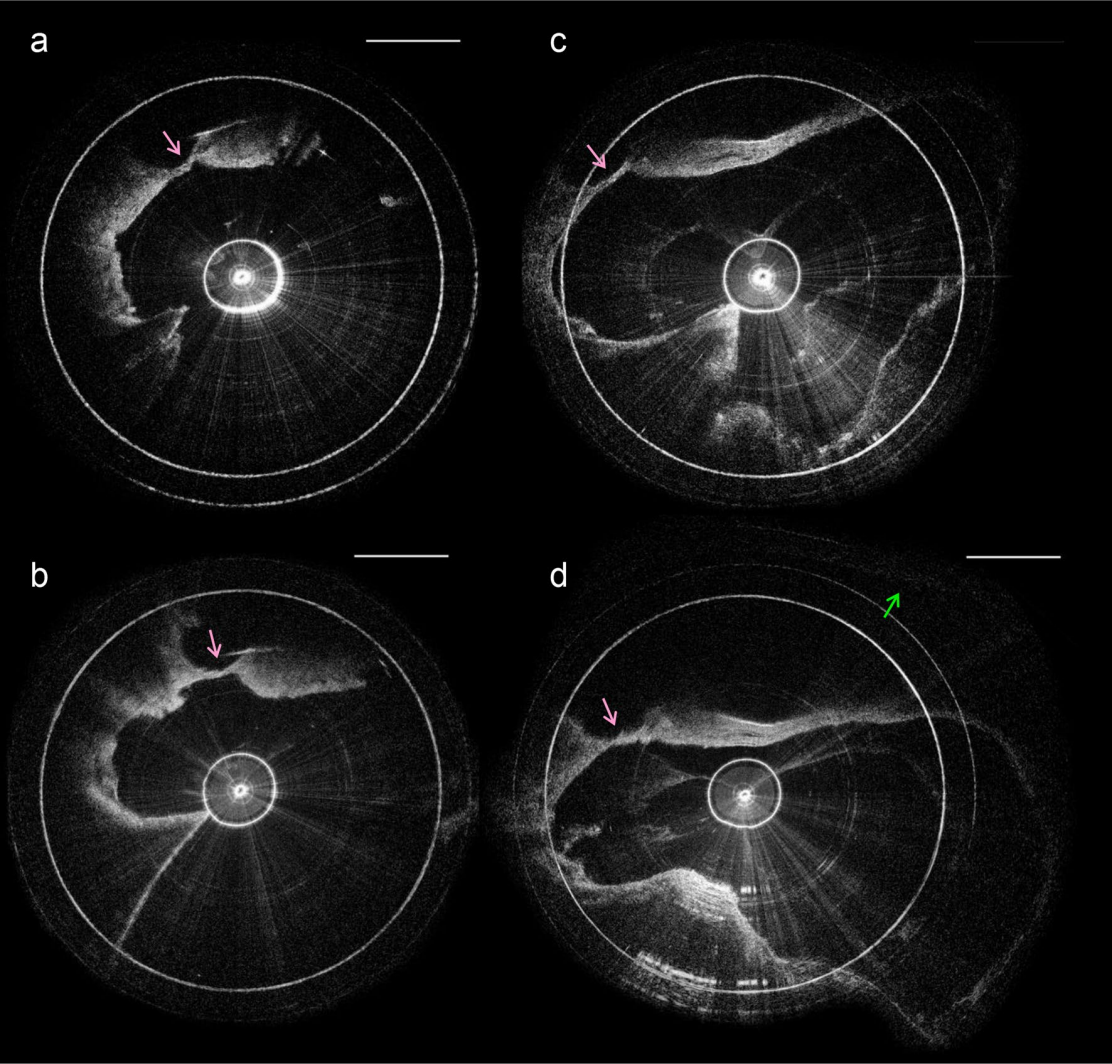
Four circularized μOCT images of the human cochlea’s interior, acquired using an intracochlear μOCT endoscope. The 2D images depicted in (**a–d**) were acquired at sequentially increasing distances from the round window. The magenta arrow in each panel indicates the location of the organ of Corti. The green arrow in panel d indicates the upper boundary of scala vestibuli. All scales = 50 μm.

**Figure 6:**
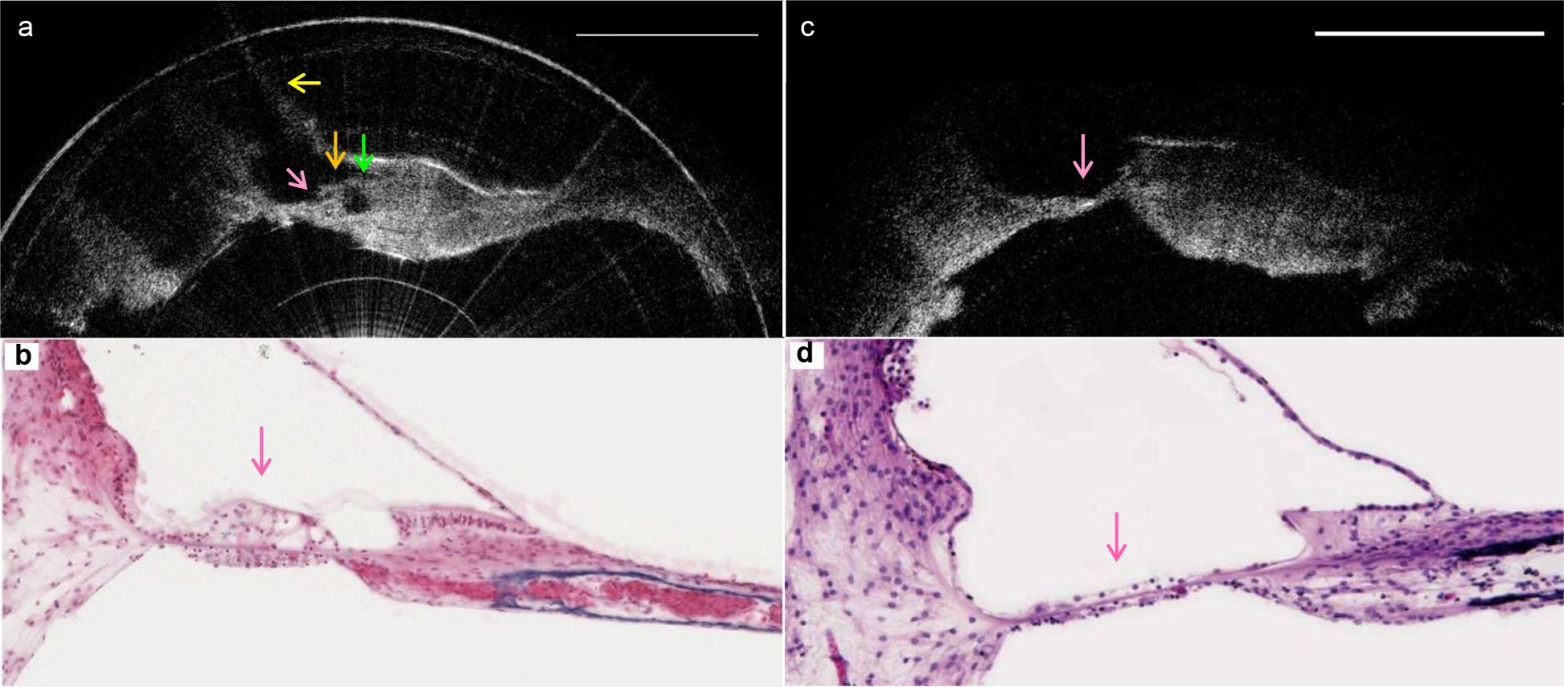
Circularized μOCT images of the human cochlea’s interior, acquired using a μOCT endomicroscope inserted through the round window and positioned within scala tympani. (**a**) Region of intact organ of Corti, showing structures that indicate the region of hair cells (magenta), the spiral limbus (green), and the tectorial (orange) and Reissner’s (yellow) membranes. (**b**) Shows a histological section acquired from a healthy region of human organ of Corti^12^ (magenta). (**c**) Hypothesized region of damaged organ of Corti (magenta) in the hook region of a human cochlea, showing flat epithelium morphology. (**d**) Shows a histological section acquired from a damaged region of human organ of Corti^12^ (magenta). All scales = 50 μm.

The current, flexible probe has a length of 2 m and an outer diameter of 0.8 mm. These dimensions facilitated insertion depths up to 8 mm via the round window (Fig. 4), corresponding to roughly the 13 kHz frequency region^11^, in three intact human temporal bone specimens that were drilled to expose the round window membrane. Cross-sectional images acquired from within scala tympani reveal this lumen’s boundaries in detail (Fig. 5). The organ of Corti was clearly visualized, and in some cases, clear distinctions between damaged and intact organ of Corti were detected (Fig. 6). In hypothesized healthy organ of Corti regions, regions that likely contained mechanosensory hair cells and the tunnel of Corti were clearly identifiable, in addition to the spiral limbus and the tectorial and Reissner’s membranes (Fig. 6a); by contrast, flat epithelium morphology was visualized in the hook region of some specimens, suggesting organ of Corti deterioration and corresponding high frequency hearing loss in these individuals (Fig. 6b). As expected, highest levels of OCT signal were visualized in the regions of the osseous spiral lamina and bone-encased lateral wall.

## Discussion

The present images are the first, to our knowledge, µOCT-based images of the organ of Corti acquired with an imaging probe inserted into scala tympani via a round window approach. Previous attempts at intracochlear endoscopy have been limited predominantly to large, rigid endoscopes that are incapable of accessing structures at depths beyond a few millimeters from the round window^13^, or have required drilling cochleostomies for insertion. Several studies on cochlear implant electrode array insertion technique have shown that a round window approach is typically significantly less invasive than a cochleostomy approach. Lin et al. previously performed intracochlear OCT endoscopy in cadaveric human temporal bone specimens and demonstrated its ability to reveal scala tympani’s boundaries, including the basilar membrane^14^; however, the authors had to perform an extended cochleostomy to acquire these images, and the image resolution was limited to 10-12 μm. By contrast, we were able to demonstrate up to 2 μm-scale resolution imaging via a minimally-invasive round window approach, and were able to see structural detail of diagnostic relevance for SNHL in the organ of Corti region. While additional high-resolution imaging techniques have been used to study temporal bones (reviewed in Bommakanti et al.^15^), such as two-photon fluorescence microscopy^16^, light sheet fluorescence microscopy^17^, synchrotron-radiation phase contrast imaging^18^, volumetric optical coherence tomography vibratometry^19^, quantitative polarized light microscopy^20^ and histology^12^, they have not yet been incorporated into a microendoscope for intracochlear applications in humans.

Results motivate further optimization of μOCT endomicroscopy for inner ear applications. We envision the first human in vivo application of μOCT endomicroscopy in patients undergoing cochlear implantation because the flexible imaging probe is of comparable diameter to the existing cochlear implant electrode arrays, and intracochlear insertion of the imaging probe would be done via the same surgical approach: trans-mastoid, trans-facial recess, round window approach. We anticipate that eventual IRB approval of first in human μOCT endomicroscopy will be smooth because intracochlear imaging would not pose a significant added risk to patients already undergoing cochlear implantation for their severe-to-profound hearing loss. In fact, visualization of intracochlear microanatomy will provide new and personalized insights into cellular correlates of cochlear implant performance; such insights are currently possible only through studies of inner ears from deceased cochlear implantees.

The primary limitations of the present design include the probe’s rigid length (approximately 3 mm), which limits insertion depth, and the helical scanning paradigm, which, due to the human cochlea’s extremely tight radius of curvature, often causes nonuniform rotational distortion (NURD)-induced image artifacts^21^. Future iterations of this design should aim to address these limitations, in addition to further improving resolution and depth-of-focus to enable more sensitive detection of cellular-level intracochlear pathologies. If successful, the development of such an imaging tool would revolutionize the study and clinical treatment of SNHL, as it would allow, for the first time, (a) direct association between inner ear pathology and hearing ability in a given patient, and thus more personalized and etiology-specific treatment recommendations, and (b) real-time investigation of scala tympani for surgical guidance during cochlear implantation. In addition, it would allow researchers to accurately evaluate patient candidacy for gene therapy trials based on the integrity of their sensory epithelia^22^, and target drug-based SNHL therapies to specific regions in the inner ear and directly visualize the effects of administration. The present results should inspire hope for patients suffering from SNHL; this technological innovation has the potential to dramatically alter the course of therapy development for human SNHL, and will inevitably increase the speed and improve the quality of research on future cures.

## Methods

### Specimen preparation

Four temporal bones were harvested from fresh human cadavers. A cylindrical saw was used to remove the portion of the skull containing the petrous temporal bone. Specimens were fixed in 10% formalin for two weeks, and were subsequently drilled to expose the round and oval windows. The bony round window niche was drilled away as necessary to allow full exposure of the round window region for probe insertion, and the round window membrane was punctured to provide a clear path for the probe. The research involving human subjects was approved by the Massachusetts General Brigham Institutional Review Board (MGB IRB), protocol title “Otopathology of the Human Temporal Bone”, protocol number 2020P000508. This MGB IRB waived the need for informed consent because the study was based on deidentified cadaveric specimens.

Six 6-week-old CBA/CaJ mice were divided into noise-exposure and control groups. Three mice (noise-exposure group) were exposed to 116 dB octave band noise (8–16 kHz) for 2 h. Temporal bones from noise-exposed and three control mice were extracted and fixed in 4% paraformaldehyde. Cochleae were subsequently extracted and the otic capsule was chipped over the basal and middle turns of the organ of Corti. The cochleae were positioned and secured under the benchtop µOCT system for imaging. All methods and experimental procedures applied on the animals were conducted in compliance with the National Institutes of Health guide for the care and use of Laboratory animals (NIH Publications No. 8023, revised 1978) and approved by the Institutional Animal Care and Use Committee (IACUC) of Massachusetts Eye and Ear Infirmary (MEEI). The study was performed in accordance with the institutional guidelines and regulations, including guidelines on Animal Research: Reporting of In Vivo Experiments (ARRIVE).

### Endomicroscope design and fabrication

The self-imaging wavefront division µOCT probe^10^ comprises a single mode fiber (630HP, Thorlabs, Newton, NJ, USA), a small segment of multimode fiber (FG050UGA, Thorlabs, Newton, NJ, USA), a spacer (FT400UMT, Thorlabs, Newton, NJ, USA), a GRIN lens (64–521, Edmund Optics, Barrington, NJ, USA), and a small custom-made mirror (Figs. 3a,b) which directed the light in a direction nearly perpendicular to the probe’s long axis. The single mode fiber, multimode fiber, and spacer components were directly spliced using a laser fusion splicer (LZM-100, AFL, Duncan, SC, USA). The small segment of multimode fiber serves as a waveguide which generates multiple propagation modes and thereby improves the system’s depth of focus while maintaining high lateral resolution^10^. The light reflected from the distal surface of the GRIN lens was leveraged as reference in realizing a common-path interferometry system configuration.

### Scanning

The µOCT probe was housed within a driveshaft to enable mechanical rotational scanning^10^. Helical scanning was achieved by coupling the probe’s proximal end to a custom-made broad-bandwidth optical rotary junction which spun the driveshaft and the optics inside, thereby scanning the beam in a circular pattern. The rotary junction was mounted onto a pullback stage for axial translation. The rotary junction speed and system frame rate were matched at roughly 17 Hz.

### Imaging system and analysis

The present spectral domain µOCT imaging system has been previously described in detail^9^ (Fig. 2a). In brief, light from a supercontinuum laser (SuperK Extreme EXR-15, NKT Photonics, Denmark) centered at 800 nm is passed through a dichroic mirror and spectral shaping filter and is then directed toward a beamsplitter that sends 10% of the light to the sample. Light backscattered from the sample and reference is recombined and guided to a spectrometer detection module comprising a telescope system, grating, focusing lens set, and a line scan camera (Basler SPL8192, Basler AG, Ahrensburg Germany). Standard OCT image reconstruction methods^23^ were used to generate depth-resolved images from the interferogram. The system has an axial point spread function with full width at half maximum of 2.5 µm in water (Fig. 3d), and a sensitivity of 90 dB. The power output at the sample was roughly 18 mW.

## References

[1] Kujawa SG, Liberman MC (2019). Translating animal models to human therapeutics in noise-induced and age-related hearing loss. Hear. Res..

[2] Kros CJ, Steyger PS (2019). Aminoglycoside- and cisplatin-induced ototoxicity: Mechanisms and otoprotective strategies. Cold Spring Harb. Perspect. Med..

[3] Finley CC (2008). Role of electrode placement as a contributor to variability in cochlear implant outcomes. Otol. Neurotol..

[4] Wanna GB (2015). Impact of intrascalar electrode location, electrode type, and angular insertion depth on residual hearing in cochlear implant patients: Preliminary results. Otol. Neurotol..

[5] Gora MJ, Suter MJ, Tearney GJ, Li X (2017). Endoscopic optical coherence tomography: Technologies and clinical applications. Biomed. Opt. Express.

[6] Hagag AM, Gao SS, Jia Y, Huang D (2017). Optical coherence tomography angiography: Technical principles and clinical applications in ophthalmology. Taiwan J. Ophthalmol..

[7] Wang J, Xu Y, Boppart SA (2017). Review of optical coherence tomography in oncology. J. Biomed. Opt..

[8] Iyer JS (2016). Micro-optical coherence tomography of the mammalian cochlea. Sci. Rep..

[9] Yin B, Hyun C, Gardecki JA, Tearney GJ (2017). Extended depth of focus for coherence-based cellular imaging. Optica.

[10] Yin B (2019). 3D cellular-resolution imaging in arteries using few-mode interferometry. Light Sci. Appl..

[11] Greenwood DD (1990). A cochlear frequency-position function for several species—29 years later. J. Acoust. Soc. Am..

[12] Merchant SN, Nadol JBJ (2010). Schuknecht’s Pathology of the Ear.

[13] Chole RA (2015). Endoscopic view of the scala tympani. Otol. Neurotol..

[14] Lin J, Staecker H, Jafri MS (2008). Optical coherence tomography imaging of the inner ear: A feasibility study with implications for cochlear implantation. Ann. Otol. Rhinol. Laryngol..

[15] Bommakanti K, Iyer JS, Stankovic KM (2019). Cochlear histopathology in human genetic hearing loss: State of the science and future prospects. Hear. Res..

[16] Yang X (2013). Two-photon microscopy of the mouse cochlea in situ for cellular diagnosis. J. Biomed. Opt..

[17] Bode J, Krüwel T, Tews B (2017). Light sheet fluorescence microscopy combined with optical clearing methods as a novel imaging tool in biomedical research. EMJ Innov..

[18] Iyer JS (2018). Visualizing the 3D cytoarchitecture of the human cochlea in an intact temporal bone using synchrotron radiation phase contrast imaging. Biomed. Opt. Express.

[19] Dong W (2018). Organ of Corti vibration within the intact gerbil cochlea measured by volumetric optical coherence tomography and vibrometry. J. Neurophysiol..

[20] Low JC, Ober TJ, McKinley GH, Stankovic KM (2015). Quantitative polarized light microscopy of human cochlear sections. Biomed. Opt. Express.

[21] Ahsen OO (2014). Correction of rotational distortion for catheter-based en face OCT and OCT angiography. Opt. Lett..

[22] Izumikawa M, Batts SA, Miyazawa T, Swiderski DL, Raphael Y (2008). Response of the flat cochlear epithelium to forced expression of Atoh1. Hear. Res..

[23] Wojtkowski M (2004). Ophthalmic imaging by spectral optical coherence tomography. Am. J. Ophthalmol..

